# Angiotensin Type 1 Receptor Antagonists Protect Against Alpha-Synuclein-Induced Neuroinflammation and Dopaminergic Neuron Death

**DOI:** 10.1007/s13311-018-0646-z

**Published:** 2018-07-09

**Authors:** Ana I. Rodriguez-Perez, Diego Sucunza, Maria A. Pedrosa, Pablo Garrido-Gil, Jaime Kulisevsky, Jose L. Lanciego, Jose L. Labandeira-Garcia

**Affiliations:** 1 Laboratory of Neuroanatomy and Experimental Neurology, Department of Morphological Sciences, Centro de Investigación en Medicina Molecular y Enfermedades Crónicas, Faculty of Medicine, University of Santiago de Compostela, 15782 Santiago de Compostela, Spain; 2 Networking Research Center on Neurodegenerative Diseases (Centro de Investigación Biomédica en Red Enfermedades Neurodegenerativas), Madrid, 28031 Spain; 3 Neurosciences Division, Centro de Investigación Médica Aplicada, University of Navarra, Pamplona, 31008 Spain; 4 Movement Disorders Unit, Neurology Department, Sant Pau Hospital and Biomedical Research Institute, Universitat Autonoma de Barcelona and Universitat Oberta de Catalunya, Barcelona, 08025 Spain

**Keywords:** Candesartan, Microglia, Neurodegeneration, Parkinson, Telmisartan, Viral vectors

## Abstract

The loss of dopaminergic neurons and α-synuclein accumulation are major hallmarks of Parkinson’s disease (PD), and it has been suggested that a major mechanism of α-synuclein toxicity is microglial activation. The lack of animal models that properly reproduce PD, and particularly the underlying synucleinopathy, has hampered the clarification of PD mechanisms and the development of effective therapies. Here, we used neurospecific adeno-associated viral vectors serotype 9 coding for either the wild-type or mutated forms of human alpha-synuclein (WT and SynA53T, respectively) under the control of a synapsin promoter to further induce a marked dopaminergic neuron loss together with an important microglial neuroinflammatory response. Overexpression of neuronal alpha-synuclein led to increased expression of angiotensin type 1 receptors and NADPH oxidase activity, together with a marked increase in the number of OX-6-positive microglial cells and expression of markers of phagocytic activity (CD68) and classical pro-inflammatory/M1 microglial phenotype markers such as inducible nitric oxide synthase, tumor necrosis factor alpha, interleukin-1β, and IL-6. Moreover, a significant decrease in the expression of markers of immunoregulatory/M2 microglial phenotype such as the enzyme arginase-1 was constantly observed. Interestingly, alpha-synuclein-induced changes in microglial phenotype markers and dopaminergic neuron death were inhibited by simultaneous treatment with the angiotensin type 1 blockers candesartan or telmisartan. Our results suggest the repurposing of candesartan and telmisartan as a neuroprotective strategy for PD.

**Electronic supplementary material:**

The online version of this article (10.1007/s13311-018-0646-z) contains supplementary material, which is available to authorized users.

## Introduction

Parkinson’s disease (PD) is the second most common neurodegenerative disorder. The loss of dopaminergic neurons and alpha-synuclein (α-syn) accumulation are major hallmarks of the disease. The etiology of PD appears multifactorial and remains largely unknown. However, regardless of the cause, it is known that oxidative stress (OS) and neuroinflammation are the major factors for progression of the disease [1–3]. Alpha-syn is a 140-amino-acid protein, predominantly located in the presynaptic terminals, and a major component of Lewy bodies. It has been associated to the regulation of neurotransmitter release, regulation of synaptic vesicles, and microtubule dynamics [4, 5]. The pathogenic role of insoluble α-syn is less known. However, microglial activation has been suggested as a major mechanism of α-syn toxicity [6].

One factor that has hampered the development of effective therapies is the lack of animal models that properly reproduce the synucleinopathy that characterizes PD reviewed in Koprich et al. [7], Sarkar et al. [8], and Visanji et al. [9]. Several models of transgenic mice overexpressing wild-type (WT) or mutated forms of human α-syn have been used to better understand the effects of α-syn accumulation [10]. However, these models were not adequate to induce a progressive and marked neurodegeneration and other major pathologic changes observed in PD. More recent models induced overexpression of α-syn using viral vectors, particularly adeno-associated viral (AAV) vectors [11, 12]. Several AAV serotypes have been used so far (AAV2, 5, and 6), although most of them have shown problems to induce adequate expression levels and significant pathology. However, more efficient AAV vector constructs led to efficient dopaminergic neuronal loss and behavioral deficits [13, 14]. An intense neuroinflammatory response was observed after induction of α-syn overexpression using AAV vectors [15, 16], which suggests that therapies that inhibit the α-synuclein-induced neuroinflammatory response may be an effective neuroprotective strategy against the progression of dopaminergic neuron degeneration.

The brain renin angiotensin system (RAS) mediates microglial polarization as angiotensin (Ang II), via its type 1 (AT1) receptor, is a major activator of the microglial NADPH oxidase complex, which enhances the neuroinflammatory response [17, 18]. Consistent with this, our previous studies showed that AT1 activation exacerbates the microglial inflammatory response, oxidative stress, and dopaminergic degeneration in 6-hydroxydopamine and MPTP models of PD, which was inhibited by AT1 receptors blockers [19–21]. In this study, we used a AAV serotype 9 (AAV9) with a small fragment of the human synapsin 1 gene promoter to restrict transgene expression exclusively to neurons [22], and we obtained stable long-term transgene expression of WT or mutated (A53T) forms of human α-syn that induced neuron degeneration in the substantia nigra pars compacta (SNpc). In this model, we investigated the α-syn-induced microglial response and its possible inhibition with AT1 receptor blockers (candesartan, telmisartan), which is particularly interesting since these drugs are currently used in clinical practice for cardiovascular diseases.

## Materials and Methods

### Experimental Design

Adult male Sprague-Dawley rats (8-10 weeks old at the beginning of the experiments; *n* = 220) were used in the present study. The animals were housed in environmentally controlled conditions: 12:12-h light/dark conditions at constant room temperature (21 ± 1 °C) with access to food and water ad libitum*.* All experiments were carried out in accordance with the European Communities Council Directive 2010/63/EU and Directive 86/609/EEC and were approved by the corresponding committee at the University of Santiago de Compostela. All surgery was performed under ketamine/medetomidine anesthesia. The rats were divided into 3 major groups. Rats in group A were injected in the right mesencephalon (close to the substantia nigra) with 2 μl of neurospecific AAV9 expressing human WT α-syn and were randomly divided into 3 subgroups: rats in subgroup A1 (*n* = 32) were simultaneously treated with vehicle, rats in subgroup A2 (*n* = 28) were simultaneously treated with the AT1 receptor antagonist candesartan, and rats in subgroup A3 (*n* = 28) were simultaneously treated with the AT1 receptor antagonist telmisartan (see below). Rats in group B were injected in the right mesencephalon (close to the substantia nigra) with 1 μl of AAV9 expressing human A53T mutated α-syn, using the procedure described above for group A. Once injected, animals were randomly divided into 3 subgroups: rats in subgroup B1 (*n* = 28) were simultaneously treated with vehicle, rats in subgroup B2 (*n* = 24) were treated with the AT1 receptor antagonist candesartan, and rats in subgroup B3 (*n* = 24) were treated with the AT1 receptor antagonist telmisartan. Rats in group C were used as controls and injected in the right mesencephalon with 2 μl of saline (group C1; *n* = 17), or neurospecific empty-null (Ф) AAV9 vectors (AAV9-null; group C2; *n* = 29), or neurospecific AAV9 expressing human green fluorescent protein (GFP) (group C3; *n* = 10). Rats from the different groups were sacrificed 1 week (*n* = 50) or 4 weeks (*n* = 170) after the nigral injection.

After the survival period, parts of the rats in the different groups (*n* = 50) were perfused and prepared for histological studies to investigate overexpression of α-syn or GFP or phosphorylated α-syn in dopaminergic neurons (TH-ir, tyrosine hydroxylase-immunoreactive), microglial cells (Iba-1-ir, ionized calcium-binding adapter molecule 1), and astrocytes (GFAP-ir, glial fibrillary acidic protein) in SNpc using double immunofluorescence and laser-scanning confocal microscopy, or histochemistry for dopaminergic cell death (TH-ir, counterstained with Nissl staining) or the microglial response (OX6-ir). In a second series of rats from different groups, brains were rapidly removed, immediately frozen in liquid nitrogen, and stored at − 80 °C until processed for determination of levels of RAS activity (AT1 receptor expression, NADPH oxidase activity), markers of phagocytic activity (CD68, cluster of differentiation 68), and markers of M1 (iNOS, inducible nitric oxide synthase; TNF-α, tumor necrosis factor alpha, interleukin (IL)-1β, IL-1β, and IL-6) and M2 (ARG-1, arginase-1) microglial phenotypes, using real-time quantitative RT-PCR, chemiluminescence, and enzyme immunoassay studies (see below).

### Synthesis and Purification of Adeno-Associated Vectors

Recombinant single-stranded AAV9 vectors were produced in our in-house facilities by a cotransfection using linear polyethylenimine 25 kDa (Polysciences, Warrington, PA) with 2 different plasmids: a plasmid containing the adenovirus helper genes, plus AAV2 rep and AAV9 cap, and a plasmid containing the transgenes of interest (either SynA53T, SynWT, eGFP, or without transgene) under the control of the human synapsin promoter, flanked by the AAV2 internal terminal repeat sequences. Viral vector particles were harvested from human embryonic kidney 293T cells (HEK293T) after 72 h posttransfection, resuspended in a lysis buffer (50 nM Tris–HCl, 150 nM NaCl, 2 mM MgCl_2_, 0.1% Triton X-100) and stored at − 80 °C until used. Viral particles were purified by ultracentrifugation in an iodoxanol gradient and were concentrated through centricon tubes (YM-100; Millipore, Bedford, MA). Viral titers were determined by quantitative PCR for viral genomic copies extracted from DBAse-treated viral particles using WPRE-directed primers (FW: CGCAACCCCACTGGTT and RV: AAAGCGAAAGTCCCGCAAAG). Obtained vector titers ranged from 1.8 × 10^12^ to 2 × 10^12^ vg/ml (viral genomes/ml).

### Stereotaxic Injections and Candesartan and Telmisartan Administration

Injections were performed in the right mesencephalon dorsal to the nigra to minimize possible traumatic damage. Stereotaxic coordinates were − 5.4 mm anterior to bregma, − 1.9 mm right of midline, and − 7.0 mm ventral to the dura; tooth bar was at − 2.3 mm [23]. The viral vectors were injected using a 5-μl Hamilton syringe coupled to a motorized injector (Stoelting, Wood Dale, IL). Injections were accomplished in pulses of 0.5 μl/min (2 μl for the AAV9-WT α-syn-injected group and 1 μl for the AAV9-A53T α-syn-injected group), and once completed, the microsyringe was left in place for an additional time of 10 min before withdrawal, to avoid viral vector reflux through the injection tract. Groups of animals received oral treatment with the AT1 receptor antagonists candesartan (groups A2 and B2 1 mg/kg/day; AstraZeneca, Madrid, Spain) or telmisartan (groups B2 and B3; 1 mg/kg/day; Sigma, St. Louis, MO) from 2 weeks before AAV9 injection until they were sacrificed. The doses were selected on the basis of the results of our previous studies [24, 25]. The powered drug was administered orally mixed with “Nocilla” hazelnut cream (Nutrexpa, Barcelona, Spain). Animals in control groups were given “Nocilla” hazelnut cream only.

### Double/Triple Immunofluorescence and Laser Confocal Microscopy

One or 4 weeks after the stereotaxic injections, rats were first transcardially perfused with 0.9% saline and then with cold 4% paraformaldehyde in 0.1 M phosphate-buffered saline, pH 7.4. The brains were removed, washed and cryoprotected in the same buffer containing 20% sucrose, and finally cut into 40-μm sections on a freezing microtome and stored in a cryoprotectant solution at − 20 °C until processing.

Double immunofluorescence labeling was performed to identify cells expressing GFP, α-syn, and phosphorylated α-syn. The antibodies against α-syn were combined with TH or GFAP or Iba-1 to study the possible colocalization with these cell type markers. Free-floating tissue sections were heated at 37 °C for 30 min in sodium citrate buffer (10 mM sodium citrate, 0.05% Tween 20, pH 6.0). After antigen retrieval, tissue sections were pre-incubated in potassium phosphate-buffered saline (KPBS)–1% bovine serum albumin (BSA) with 5% normal donkey serum (Sigma) for 60 min at room temperature and then incubated overnight at 4 °C in the corresponding mix of primary antibodies raised against α-synuclein (mouse IgG, 1:500, ab80627, Abcam, Cambridge, England, UK, for WT synuclein; mouse IgG, 1:200, 18-0215, Invitrogen, Paisley, UK, for A53T synuclein), phosphorylated α-syn (rabbit IgG, 1:1000, ab51253, Abcam), TH (rabbit IgG, 1:1000 ab152, Millipore; or mouse IgG, 1:10,000, T2928, Sigma), GFAP (mouse IgG, 1:500; MAB360, Millipore), and Iba-1 (rabbit IgG, 1:500, 019-19741, Wako, Richmond, VA) diluted in KPBS–1% BSA with 4% normal donkey serum. Finally, the immunoreaction was visualized with the corresponding fluorescent secondary antibodies: Alexa Fluor 568-conjugated donkey anti-rabbit IgG (1:200, Molecular Probes, Eugene, OR) and Alexa Fluor 488-conjugated donkey anti-mouse IgG (1:200, Molecular Probes). Finally, tissue sections were incubated for 30 min at RT with the DNA-binding dye Hoechst 33342 (3 × 10^−5^ M, Sigma), in KPBS, mounted on gelatin-coated slides and coverslipped with Shandon Immu-Mount (Thermo fisher Scientific, Waltham, MA). Tissue sections were visualized using a confocal laser scanning microscope (AOBS-SP5X; Leica Microsystems Heidelberg GmbH, Mannheim, Germany). For colocalization analysis, 3 images of the substantia nigra per animal were obtained at different coordinates of its anteroposterior axis (− 5.20, − 5.60, and − 6.04 mm from bregma). Images were obtained by laser scanning microscopy with the aid of a × 10 objective and using constant microscope parameters and similar laser intensity. The substantia nigra was delimited in each image and the rate of colocalization of TH and P(Ser129)-syn was measured by using the LAS AF Lite software (Leica).

### Western Blot

For Western blot analysis of α-syn and phosphorylated α-syn, the nigral region in the ventral mesencephalon was dissected and the tissue was homogenized in radioimmunoprecipitation assay buffer containing protease inhibitor cocktail (P8340, Sigma), phenylmethylsulfonyl fluoride (P7626, Sigma), and phosphatase inhibitor cocktail (P5726, Sigma). For determination of α-synuclein, the resulting homogenates were centrifuged for 20 min at 12,000×*g*. For phosphorylated α-synuclein, samples were processed as previously described [14] with slight modifications. Briefly, homogenates were incubated for at least 1 h in ice, then samples were centrifuged for 30 min at 120,000×*g* and the resulting supernatants were recollected. Both for α-synuclein and phosphorylated α-synuclein, protein concentrations were determined by the Bradford protein assay. Equal amounts of protein (35 μg) were separated by 5 to 10% Bis–Tris polyacrylamide gel and transferred to nitrocellulose membrane. Then, the membranes were treated with 0.4% PFA for 30 min at room temperature before blocking them with phosphoblocker (10%, AKR-103, Cell Biolabs, San Diego, CA). The membranes were incubated overnight with primary antibodies against α-synuclein (1:1000, ab138501, Abcam) or phosphorylated α-synuclein (1:1000, ab51253, Abcam). The HRP-conjugated secondary antibody used was mouse anti-rabbit (1:2500, sc-2357, Santa Cruz Biotechnology, Dallas, TX). Immunoreactivity was detected with an Immun-Star HRP Chemiluminescent Kit (170-5044, Bio-Rad; Hercules, CA) and visualized with a chemiluminescence detection system (Molecular Imager ChemiDoc XRS System, Bio-Rad). Blots were stripped and reprobed for anti-glyceraldehyde 3-phosphate dehydrogenase (GAPDH) (1:25,000, G9545, Sigma) as loading control.

### Immunohistochemistry, Nissl Staining, and Stereological Analysis

TH and OX6 staining were used to label dopaminergic cells and classically activated microglia, respectively. The mouse monoclonal MHC class II antibody (OX6) was selected because microglial MHCII expression has been shown to be strikingly induced by α-syn *in vitro* and *in vivo*, and it is known that the expression of MHCII plays a major role in both the innate and the adaptive immune response [26]. Sections were incubated for 1 h in 10% normal serum with 0.25% Triton X-100 in 20 mM KPBS containing 1% BSA (KPBS–BSA), then incubated overnight at 4 °C with mouse monoclonal antiserum to TH (1:10,000, T2928, Sigma) or mouse monoclonal MHC class II antibody (OX6, 1:50, MCA46G, Bio-Rad) at 4 °C in 20 mM KPBS containing 1% BSA, 2% normal serum, and 0.25% Triton X-100. The following day, the sections were incubated, firstly for 90 min with the corresponding biotinylated secondary antibodies (1:200), and then for 90 min with an avidin–biotin–peroxidase complex (1:50, Vector, Burlingame, CA). Finally, the labeling was visualized with 0.04% hydrogen peroxide and 0.05% 3,3′-diaminobenzidine (Sigma), containing 0.1% nickel sulfate to intensify the microglial staining. For negative control staining, sections were incubated in media lacking primary antibodies. In order to confirm dopaminergic cell death and not just phenotypic downregulation in TH activity, series of sections through the entire substantia nigra of control and treated rats were counterstained with Cresyl violet. Brain sections were mounted on gelatin-coated glass slides and stained for 5 min with 1% Cresyl violet (C0775, Sigma) dissolved in distilled water. Then, stained sections were dehydrated through graded ethanol (100%, 95%, 70%, and 50%), cleared in xylene for 5 min, covered with DPX mounting medium (Panreac Applichem, Barcelona, Spain), and coverslipped. Finally, the total number of neurons in the SNc was estimated using the unbiased stereology method (see below).

The total number of TH-ir neurons and OX6-ir density in the substantia nigra was estimated by an unbiased stereological method (the optical fractionator) using an Olympus CAST-Grid system (Computer Assisted Stereological Toolbox; Olympus, Ballerup, Denmark). Uniform randomly chosen sections through the SNpc (every fourth section) from the different groups were analyzed for the total number of TH-ir or OX6-ir cells by means of a stereological grid (fractionator), and the nigral volume was estimated according to Cavalieri’s method [27]. To confirm that overexpression of α-syn promoted dopaminergic neuron death, series of sections through the entire SNpc of control and treated rats were counterstained with Cresyl violet, and the total number of neurons in the SNpc was estimated by the unbiased stereology method described above for TH-ir cells. Neurons were distinguished from glial cells on a morphological basis, and neurons with visible nuclei were counted as above for TH-ir neurons. For details, see Rey et al. [20].

The density of striatal dopaminergic terminals was estimated as the optical density of the striatal TH-ir with the aid of NIH-Image 1.55 image analysis software (National Institutes of Health, Bethesda, MD) in a personal computer coupled to a videocamera (CCD-72, DAGE-MTI, INC, Michigan City, IND) and a constant illumination light table (Northern Light, St. Catharines, Canada). At least 4 sections through the central striatum of each animal were measured, and for each section, the optical densities were corrected by subtraction of background, as observed in the corpus callosum.

### High Performance Liquid Chromatography

Dopamine (DA) and its metabolites 3,4-dihydroxyphenylacetic acid (DOPAC) and homovanilic acid (HVA) were measured in the striata by high-performance liquid chromatography (HPLC) analysis, as previously described [28]. Briefly, rat striatal tissue was homogenized and centrifuged (14,000×*g* for 20 min at 4 °C). Then, the supernatant fractions were filtered and injected directly into the HPLC (Shimadzu LC prominence, Shimadzu Corporation, Kyoto, Japan). DA, DOPAC, and HVA were separated on a reverse phase analytical column (Waters Symmetry300C18; Waters, Milford, MA). The mobile phase (pH 4) consisted of 10% MeOH, 70 mM KH_2_PO_4_, 1 mM octanesulfonic acid, and 1 mM EDTA and was delivered at a rate of 1 ml/min. Detection was performed with a coulometric electrochemical detector (ESA Coulochem III; ESA, Chelmsford, MA). Data were acquired and processed with the Shimadzu LC solution software (Shimadzu Corporation) and were expressed as nanogram per milligram of wet tissue. The DOPAC/DA ratio was calculated for each animal as an index of the DA turnover.

### Motor Behavioral Analysis: Cylinder Test, Rotarod Test, and Locomotor Activity

Spontaneous forelimb use was assessed in the cylinder test [29]. The rats were placed in a glass cylinder (20 cm in diameter), and forelimb use was analyzed by videotaping the animals as they moved freely in the cylinder and explored the environment. Mirrors were placed behind the cylinder to allow observation of the animals when they were turned away from the camera. An observer blinded to the identity of the animals scored the number of forelimb contacts with the cylinder wall. In rats with unilateral dopaminergic denervations, forelimb asymmetry is expressed as the use of the impaired paw and expressed as the percentage of the total number of touches (20 touches were counted for each animal). An unbiased control animal would thus receive a score of 50%, whereas lesions usually reduce performance of the impaired paw in this test.

Our original rotarod test [30, 31] was performed with slight modifications [32], using an automated 4-lane rotarod unit (CR-1 Rotamex System, Columbus Instruments, Columbus, OH). A session protocol with an accelerated rotational speed was used. Animals were pretrained during 2 consecutive days to reach a stable performance in the test. For rotarod testing, the rats were placed on the rod at an initial speed of 4 rpm. Then, the rod speed was increased to 44 rpm over 90 s, and the total running time on the rod was recorded. In all cases, tests were performed on 3 consecutive days, and the results were averaged to obtain a single value for each rat. Finally, integrated measures for the rotarod performance of each rat were obtained as the area under the curve (AUC).

Spontaneous locomotor activity was automatically monitored with the aid of a Videomex-X motion analyzer (Columbus Instruments), which is a video-based apparatus that monitors the video image in real time (20 frames per second) [33]. The multiple motion monitor software option was used to estimate total activity (rate of motion) of each group of animals. The rate of motion consists of the amount that the picture changes from 1 frame to the next. The program counts the number of pixel changes caused by the movements of all the animals in the picture. Each group of animals was acclimatized to the Videomex for at least 10 min, and locomotor activity was monitored for 30 min. All trials were carried out in a black-walled open-topped box of 70 × 75 × 40 cm. Data obtained from 3 consecutive days were used for the analysis and expressed as percentage of the control group.

### RNA Extraction and Real-Time Quantitative Reverse Transcriptase–Polymerase Chain Reaction

Total RNA from the nigral region was extracted with TRIzol (Invitrogen), according to the manufacturer’s instructions; 2.5 μg of RNA was reverse-transcribed to complementary DNA with deoxynucleotide triphosphates, random primers, and Moloney murine leukemia virus reverse transcriptase (200 U; Invitrogen). Real-time polymerase chain reaction was used to examine relative levels of iNOS and ARG-1 messenger RNA. Experiments were performed with a real-time iCycler polymerase chain reaction platform (Bio-Rad). GAPDH was used as a housekeeping gene and was amplified in parallel with the genes of interest. The comparative cycle threshold values (Ct, cycle threshold) method (2^−ΔΔCt^) was used to examine the relative mRNA expression. A normalized value is obtained by subtracting Ct of GAPDH from Ct of the interest (ΔCt). As it is uncommon to use ΔCt as relative expression data due to this logarithmic characteristic, the 2^−ΔΔCt^ parameter was used to express the relative expression data [34, 35]. Finally, the results were expressed as mean values ± SEM. Primer sequences were as follows: for iNOS, forward 5′-AAGCTGCATGTG ACTCCAT-3′, reverse 5′-GGTGAAGGGTGTCGTGAA-3′; for ARG-1, forward 5′-ATATCTGCCAAGGACATCGT-3′, reverse 5′-TCCACTTCAGTCATTGAGAAATAC-3′; for AT1, forward 5′-TTCAACCTCTACGCCAGTGTG-3′, reverse 5′-GCCAAGCCAGCCATCAGC-3′; for CD68, forward 5′-AGACGACAATCAACCTACC-3′, reverse 5′-ATGCTGAAGAAATGAGGA-3′; for IL-1β, forward 5′-ATCTCACACAGCAGCATCTC-3′, reverse 5′-TAGCAGGTCGTCATCATC-3′; for IL-6, forward 5′-CAGCCAGTTGCCTTCTTG-3′, reverse 5′-CCTCTGTGAAGTCTCCTCTC-3′; and for β-actin, forward 5′-TCGTGCGTGACATTAAAGAG-3′, reverse 5′-TGCCACAGGATTCCATACC-3′.

### TNF-α Quantification

Tissue from rat ventral midbrain was homogenized in RIPA buffer containing protease inhibitor cocktail (P8340, Sigma) and phenylmethylsulfonyl fluoride (P7626, Sigma). The homogenates were centrifuged at 12,000×*g* for 20 min at 4 °C, and the protein concentrations were determined by the Bradford protein assay. The levels of TNF-α were quantified with rat specific enzyme-linked immunosorbent assay kits according to the manufacturers’ instructions (rat TNF-α from Diaclone 865.000.96, Gen-Probe Diaclone SAS, Besançon, France). Finally, TNF-α content was obtained in picograms per milliliter protein and expressed as percentages of the content in the control group samples.

### NADPH Oxidase Activity

NADPH oxidase activity in ventral mesencephalic tissue was measured by lucigenin-enhanced chemiluminescence with an Infinite M200 multiwell plate reader (TECAN, Salzburg, Austria), as previously described [36, 37]. Chemiluminescence was expressed as relative light units (RLU/min/mg protein).

### Statistical Analysis

Statistical analyses were carried out with SigmaStat 3.0 from Jandel Scientific (San Rafael, CA). All values are expressed as mean ± SEM (*n* = 4-6). Differences among means were analyzed using one-way ANOVA followed by a post hoc Holm–Sidak test. The normality of populations and homogeneity of variances were tested before each ANOVA. In all analyses, the null hypothesis was rejected at 0.05 level.

## Results

### Transgene Expression Following AAV Vector Injection

**Fig. 1:**
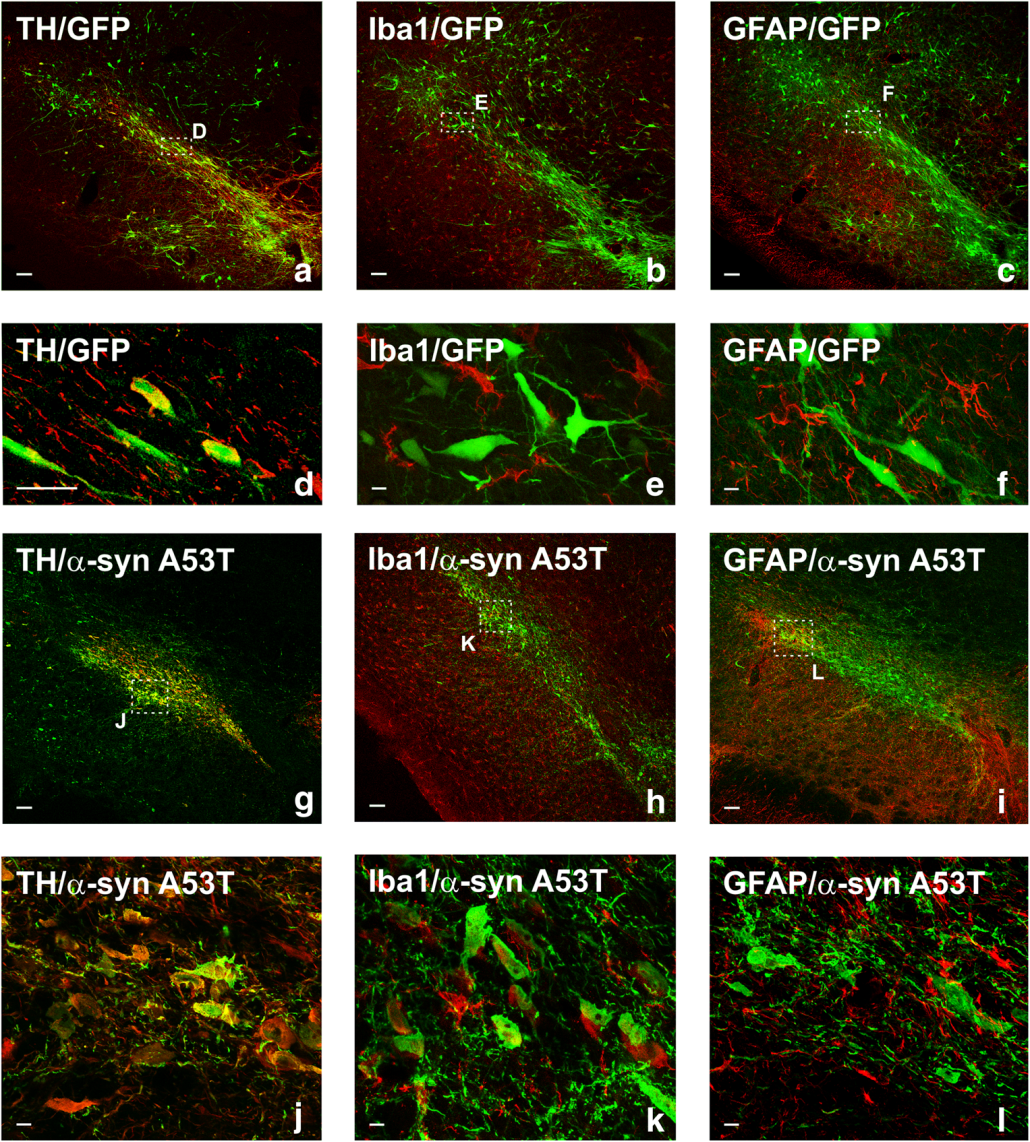
Double immunofluorescence for the dopaminergic marker TH (a, d, g, j), or the microglial marker Iba-1 (b, e, h, k), or the astrocytic marker GFAP (c, f, i, l) (red) and different transgenes (GFP: a–f; α-syn-A53T: g–l) in the substantia nigra region 1 week after the injection of AAV9 expressing human GFP or human α-syn-A53T, respectively (green). Laser confocal microscopy shows colocalization (yellow) of GFP or α-syn-A53T with the dopaminergic marker TH (a, d, g, j). However, GFP or α-syn-A53T is not expressed in microglia or astrocytes. Areas squared in (a)–(c) and (g)–(i) are magnified in (d)–(f) and (j)–(l), respectively. Scale bars = 75 μm (a–c, g–i), 25 μm (d), and 7.5 μm (e–f, j–l). Abbreviations: AAV9 = adeno-associated viral vectors serotype 9; α-syn A53T = A53T mutated alpha-synuclein; GFAP = glial fibrillary acidic protein; GFP = green fluorescent protein; Iba-1 = ionized calcium-binding adapter molecule 1; TH = tyrosine hydroxylase

Double immunofluorescence and laser confocal microscopy revealed that AAV9-vector injection induced overexpression of GFP or α-syn in dopaminergic neurons, but not in microglia and astrocytes in rat SNpc. One week after injection, we observed widespread labeling for GFP or α-syn, which colocalized with the dopaminergic marker TH showing efficient transduction of the nigral dopaminergic neurons. In keeping with our previous findings [38], we did not find any significant colocalization between α-syn or GFP and the microglial population marker Iba-1 or the astrocyte marker GFAP. In addition, TH-negative (i.e., nondopaminergic) neurons were also labeled (Figs. 1 and 3a, h).

**Fig. 2:**
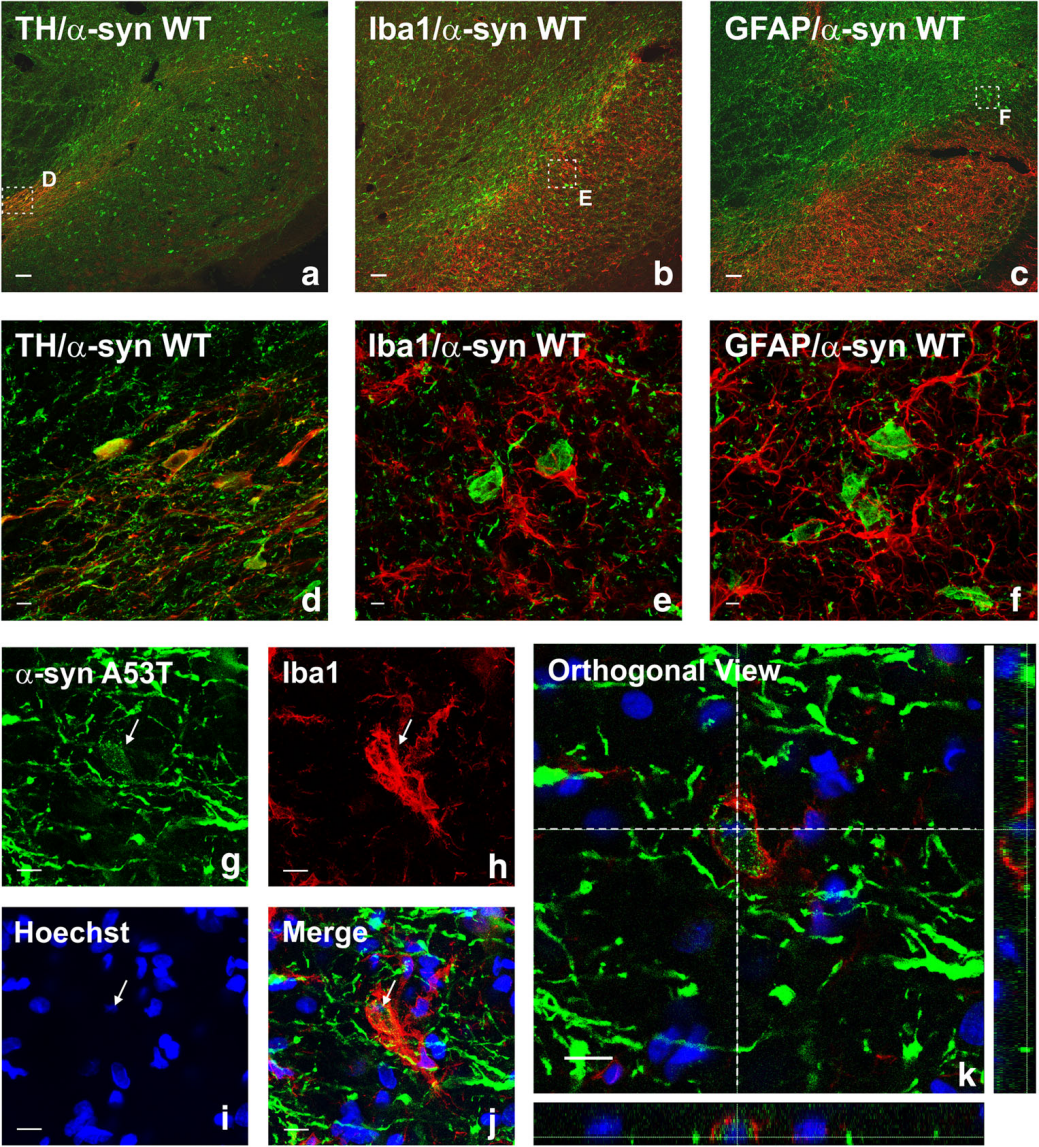
Double and triple immunofluorescence for (red) the dopaminergic marker TH (a, d), or the astrocytic marker GFAP (c, f), or the microglial marker Iba-1 (b, e, g–k), or the nuclear marker Hoechst 33342 (i–k; blue), and different transgenes (green; α-syn-WT: a–f; α-syn-A53T: g–k) in the substantia nigra region 4 weeks after the injection of AAV9 expressing human WT α-syn (a–f) or human α-syn-A53T (g–k), respectively. Laser confocal microscopy shows colocalization (yellow) of α-syn with the dopaminergic marker TH (a, d). However, α-syn is not expressed in microglia or astrocytes. Areas squared in (a)–(c) are magnified in (d)–(f). An intense microglial activation is observed in (b) and (e). A microglial cell engulfing α-syn-A53T from a degenerating neuron (arrow) is shown in (g)–(k). Scale bars = 75 μm (a–c), 7.5 μm (d), 5 μm (e–f), and 10 μm (g–k). Abbreviations: α-syn A53T = A53T mutated alpha-synuclein; α-syn WT = wild-type alpha-synuclein; GFAP = glial fibrillary acidic protein; Iba-1 = ionized calcium-binding adapter molecule 1; TH = tyrosine hydroxylase

**Fig. 3:**
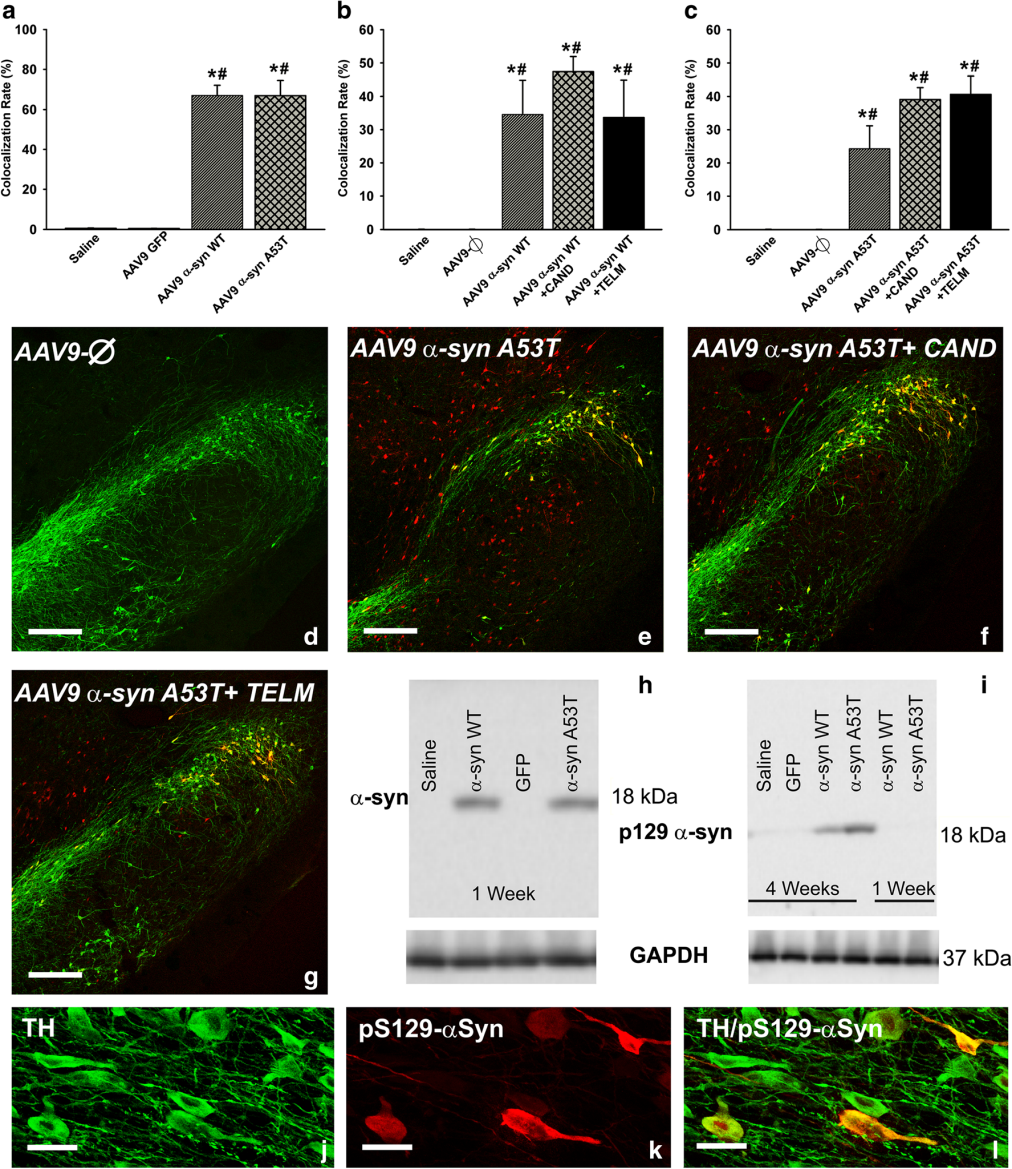
Expression of α-syn (a, h) and phosphorylated α-syn (b–g, i–l) 1 week (a, h) and 4 weeks (b–g, i–l) after injection of AAV9 vectors. The rate of expression of α-syn in dopaminergic neurons (TH-ir) of rats injected with AAV9-α-syn WT or AAV9-α-syn A53T is shown in (a). The rate of expression of phosphorylated α-syn in dopaminergic neurons (TH-ir) of rats injected with AAV9-α-syn WT or AAV9-α-syn A53T and treated or not treated with candesartan or telmisartan is shown in (b) and (c). The colocalization (yellow) of phosphorylated α-syn (red) and dopaminergic neurons (green) in the different experimental groups is illustrated in (d)–(g) and magnified in (j)–(l). The expression of α-syn 1 week after injection (h) and phosphorylated α-syn in the nigral region (i.e., dopaminergic and nondopaminergic cells) 1 and 4 weeks after injection (i) was confirmed by Western blot. Data are means ± SEM. *p < 0.05 relative to the group injected with saline; ^#^*p* < 0.05 relative to the AAV9-Ф-injected group. One-way ANOVA followed by Holm–Sidak post hoc test. Scale bars = 250 μm (d–g) and 25 μm (j–l). Abbreviations: α-syn A53T = A53T mutated alpha-synuclein; α-syn WT = wild-type alpha-synuclein; ANOVA = analysis of variance; CAND = candesartan; SEM = standard error of the mean; TELM = telmisartan; Ф = empty-null

Four weeks after injection, a clear loss of TH-positive cells was observed in the SNpc of the lesioned groups. Despite the intense microglial reaction observed in the ipsilateral ventral mesencephalon (see below), labeling for α-syn was located in neurons (Fig. 2a–f). However, some microglial phagocytic cells showed engulfed α-syn (Fig. 2g–k). The pattern of SNpc labeling and colocalization with dopaminergic neurons and glial cells was similar for WT α-syn and A53T mutated α-syn. Furthermore, intense labeling for phosphorylated α-syn was observed in the surviving dopaminergic neurons in the lesioned groups (Fig. 3b–g, i–l). Interestingly, the rate of phosphorylated α-syn/TH was not decreased in rats treated with AT1 blockers in comparison with untreated rats, which suggests that a decrease in α-syn phosphorylation is not a major mechanism responsible for the neuroprotective effects of candesartan and telmisartan described below (Fig. 3b, c).

### Loss of Dopaminergic Neurons After Overexpression of WT or Mutated A53T Alpha-Synuclein: Effect of AT1 Receptor Blockers

**Fig. 4:**
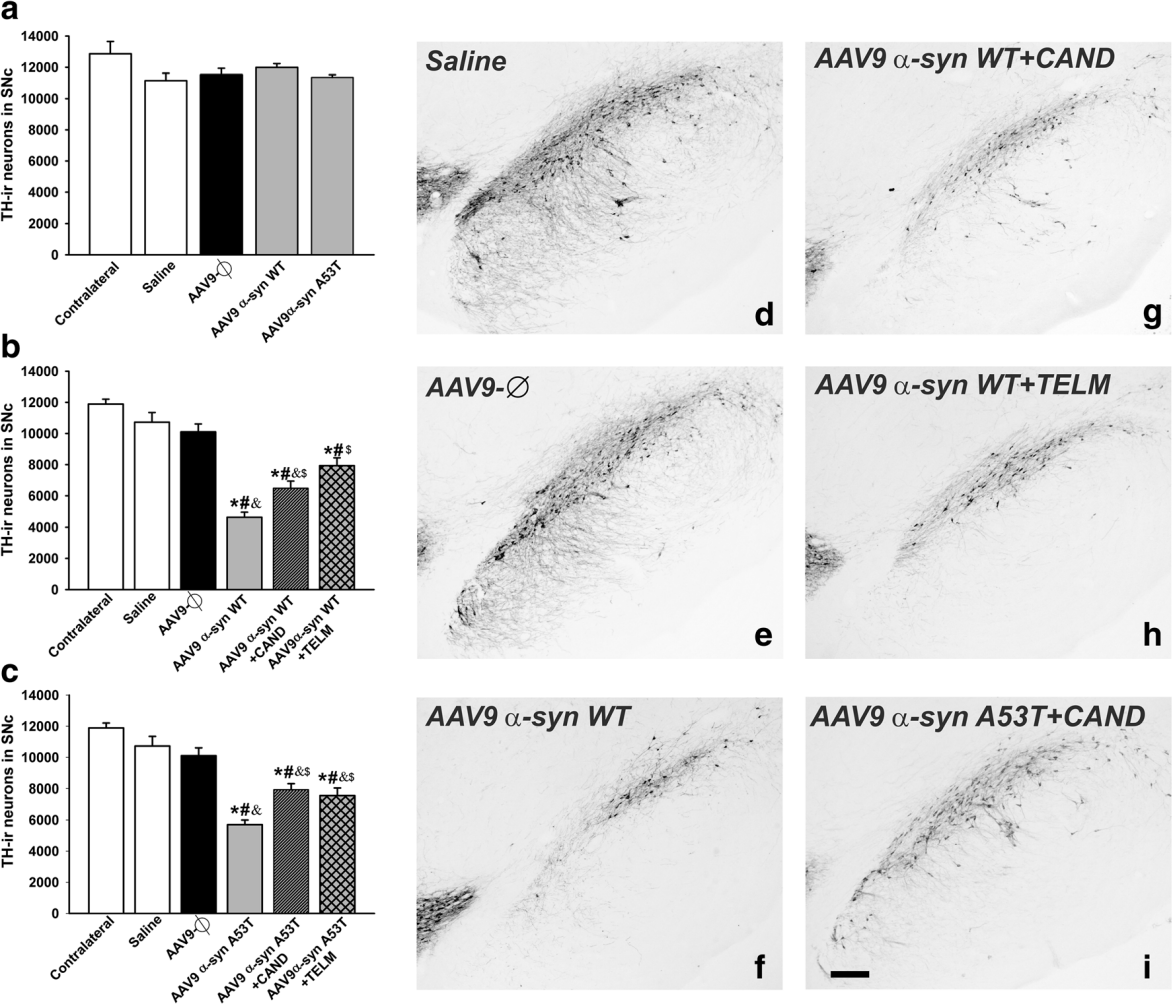
Dopaminergic (TH-ir) neurons in the substantia nigra compacta (SNc) of the noninjected side (contralateral, control) and 1 week (a) or 4 weeks (b–i) after injection of saline, or empty AAV9 vectors (AAV9-Ф), or AAV9 expressing human WT α-syn, or AAV9 expressing human A53T mutated α-syn in rats not treated or treated with the AT1 antagonist candesartan (CAND) or telmisartan (TELM). Representative photomicrographs of the substantia nigra compacta of different groups of rats are shown in (d)–(i). The estimated total number of dopaminergic (TH-ir) neurons in the substantia nigra compacta of the different experimental groups is shown in (a)–(c). Data are means ± SEM. ^*^p < 0.05 relative to the noninjected side (contralateral, control), ^#^*p* < 0.05 relative to the group treated with saline, ^&^*p* < 0.05 relative to the AAV9-Ф-injected group, ^$^*p* < 0.05 relative to the group injected with AAV9 expressing human WT α-syn (b) or expressing human A53T mutated α-syn (c). One-way ANOVA and Holm–Sidak post hoc test. Scale bar = 200 μm. Abbreviations: ANOVA = analysis of variance; SEM = standard error of the mean; Ф = empty-null

**Fig. 5:**
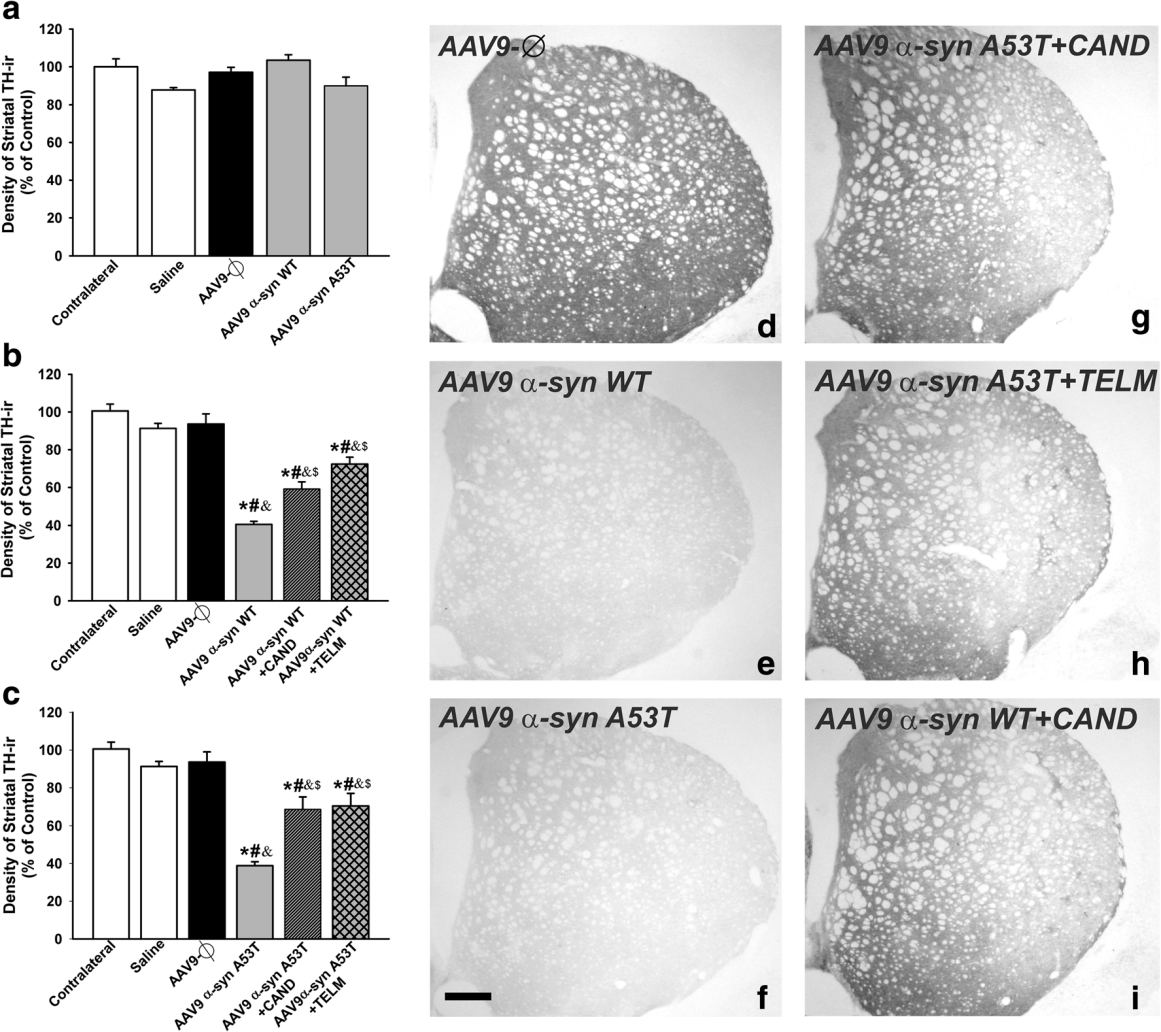
Optical densitometry of TH-ir striatal dopaminergic terminals of the noninjected side (contralateral, control) and 1 week (a) or 4 weeks (b–i) after injection of saline, or empty AAV9 vectors (AAV9-Ф), or AAV9 expressing human WT α-syn, or AAV9 expressing human A53T mutated α-syn in rats not treated or treated with the AT1 antagonists candesartan (CAND) or telmisartan (TELM). Representative photomicrographs of the striatal TH immunoreactivity of different groups of rats are shown in (d)–(i). The optical densitometry of striatal TH-ir of the different experimental groups is shown in (a)–(c). Data are means ± SEM. ^*^p < 0.05 relative to the noninjected side (contralateral, control), ^#^*p* < 0.05 relative to the group treated with saline, ^&^*p* < 0.05 relative to the AAV9-Ф-injected group, ^$^*p* < 0.05 relative to the group injected with AAV9 expressing human WT α-syn (b) or expressing human A53T mutated α-syn (c). One-way ANOVA and Holm–Sidak post hoc test. Scale bar = 500 μm. Abbreviations: ANOVA = analysis of variance; SEM = standard error of the mean; Ф = empty-null

Consistent with that observed in previous studies with similar animal models [13, 14], no appreciable loss of dopaminergic neurons was observed (Fig. 4a) 1 week after injection. No significant changes were observed in striatal TH-ir, suggesting no significant loss of striatal dopaminergic terminals (Fig. 5a).

Four weeks after injection, a total number of 10,725 ± 621 (mean ± SEM) TH-positive neurons were counted in the ipsilateral SNpc of saline-injected rats, which was not significantly different from that observed in AAV9-α-syn-Ф-injected animals (10,107 ± 503 neurons). Injection of AAV9-WT α-syn induced a loss of TH-ir neurons of about 57% (i.e., 4641 ± 329) (Fig. 4b, d–f). The loss of neurons in the SNpc (not just TH downregulation) was confirmed by counting neurons in Nissl-stained sections. We observed 12,915 ± 312 Nissl-stained neurons in controls and 11,397 ± 605 neurons in animals injected with empty vectors. However, 5019 ± 340 Nissl-stained neurons were counted in the AAV9-WT α-syn-injected group. Similarly, injection of AAV9-A53T α-syn induced a marked loss of Nissl-stained neurons (6409 ± 342) in comparison with control rats or rats injected with AAV9 empty vectors (Fig. 4c). In addition, a loss of dopaminergic striatal terminals was confirmed by optical density of striatal TH-ir both in rats injected with AAV9-WT α-syn and rats injected with AAV9-A53T α-syn, which showed a significant decrease in striatal TH-ir relative to control rats or rats injected with AAV9 empty vectors (Fig. 5b–f).

Rats injected with AAV9-WT α-syn and treated with the AT1 receptor antagonist candesartan showed a significant loss of dopaminergic neurons (6484 ± 460; about 40% decrease in comparison with controls) and striatal dopaminergic terminals; however, the neuron loss and striatal loss of dopaminergic terminals were significantly less than those observed in rats not treated with candesartan. A similar neuroprotective effect was observed in AAV9-WT α-syn rats treated with telmisartan (6484 ± 460 neurons; about 40% decrease in the number of nigral neurons and the corresponding striatal dopaminergic terminals relative untreated controls). Similar neuroprotective effects were observed in rats injected with AAV9-A53T α-syn and treated with candesartan or telmisartan (Figs. 4b, c, g–i and 5b, c, g–i). We also counted Nissl-stained neurons to confirm the neuroprotective effects of candesartan, showing a number of neurons (7066 ± 475 neurons in the group injected with AAV9-WT α-syn; 8583 ± 518 in the group injected with AAV9-A53T α-syn), which was significantly higher than in lesioned and untreated rats (5019 ± 340 in the group injected with AAV9-WT α-syn; 6409 ± 342 in the group injected with AAV9-A53T α-syn). Nissl-stained neuron counts also confirmed the neuroprotective effects of telmisartan, showing a number of neurons (8432 ± 494 neurons in the group injected with AAV9-WT α-syn; 8523 ± 530 in the group injected with AAV9-A53T α-syn), which was significantly higher than in lesioned and untreated rats (see above). No significant differences were found between rats injected with AAV9-WT α-syn and treated with candesartan and rats injected with AAV9-WT α-syn and treated with telmisartan.

### HPLC Analysis and Motor Behavior

**Fig. 6:**
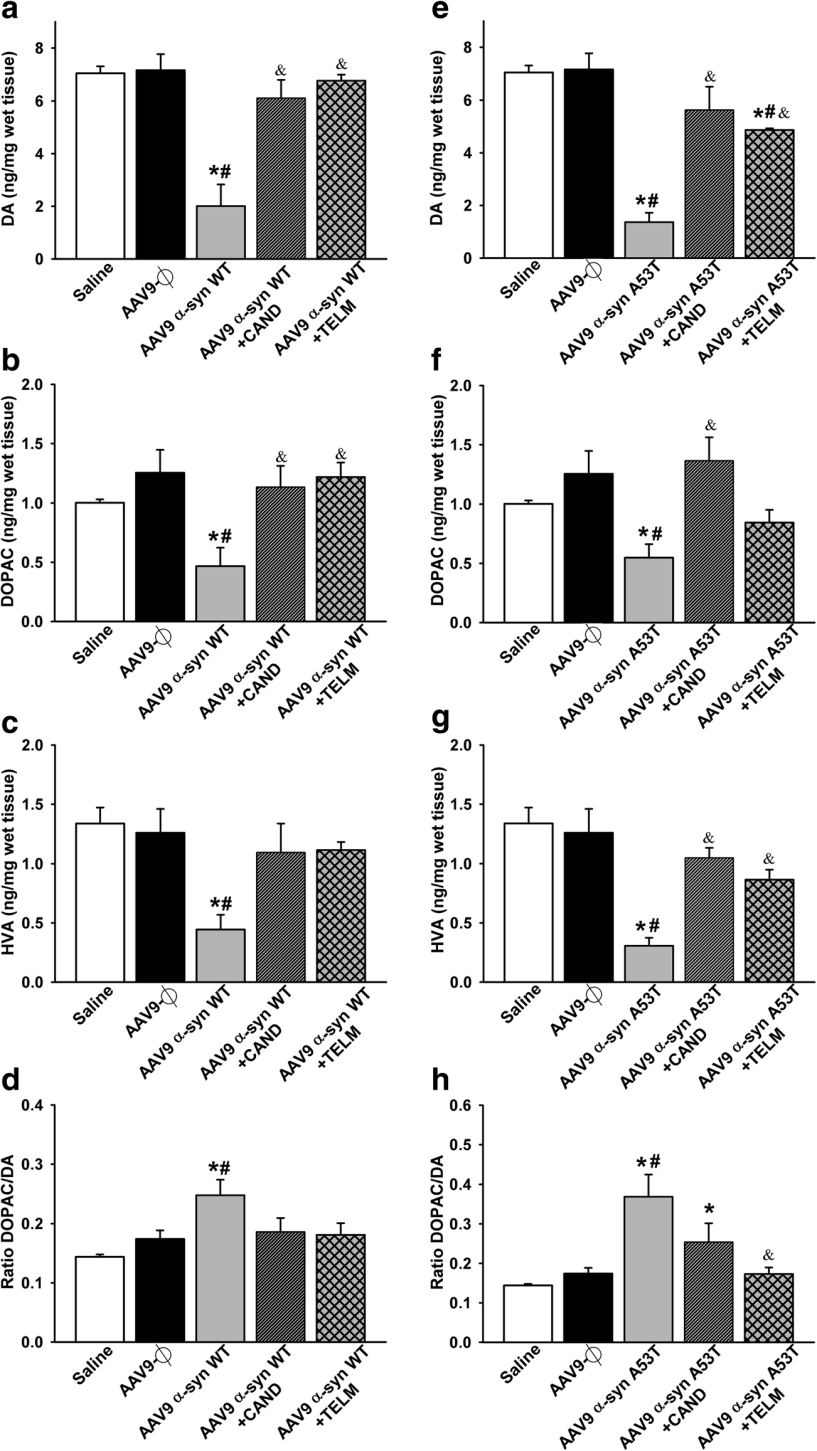
HPLC analysis of the striatal levels of DA (a, e), DOPAC (b, f), and HVA (c, g) 4 weeks after injection of AAV9 vectors in control groups and groups injected with AAV9-α-syn WT (a–d) or AAV9-α-syn A53T (e–h). The striatal turnover of DA calculated by the DOPAC/DA ratio is also shown (d, h). Measurements show significant reductions in DA and metabolites in lesioned rats relative to controls (saline and empty vectors), which were significantly improved by treatment with candesartan or telmisartan. Data are means ± SEM. ^*^p < 0.05 relative to the saline-injected group; ^#^*p* < 0.05 relative to the AAV9-Ф-injected group; ^&^*p* < 0.05 relative to the group injected with AAV9 expressing human WT α-syn (a, b) or expressing human A53T mutated α-syn (e–h). One-way ANOVA followed by Holm–Sidak post hoc test. Abbreviations: α-syn A53T = A53T mutated alpha-synuclein; α-syn WT = wild-type alpha-synuclein; ANOVA = analysis of variance; CAND = candesartan; DA = dopamine; DOPAC = 3,4-dihydroxyphenylacetic acid; HVA = homovanilic acid; SEM = standard error of the mean; TELM = telmisartan; Ф = empty-null

Four weeks after injection, consistent with the loss of dopaminergic neurons in the SNpc and the loss of striatal terminals, HPLC analysis revealed a marked decrease in the levels of dopamine and metabolites (DOPAC and HVA) in rats injected with AAV9-WT α-syn (Fig. 6a–d) and rats injected with AAV9-A53T α-syn (Fig. 6e–h) in comparison with controls. In addition, we observed an increase in the DOPAC/DA ratio that suggested an increase in DA turnover. Interestingly, the levels of dopamine and metabolites significantly increased in lesioned rats treated with candesartan or telmisartan (Fig. 6).

**Fig. 7:**
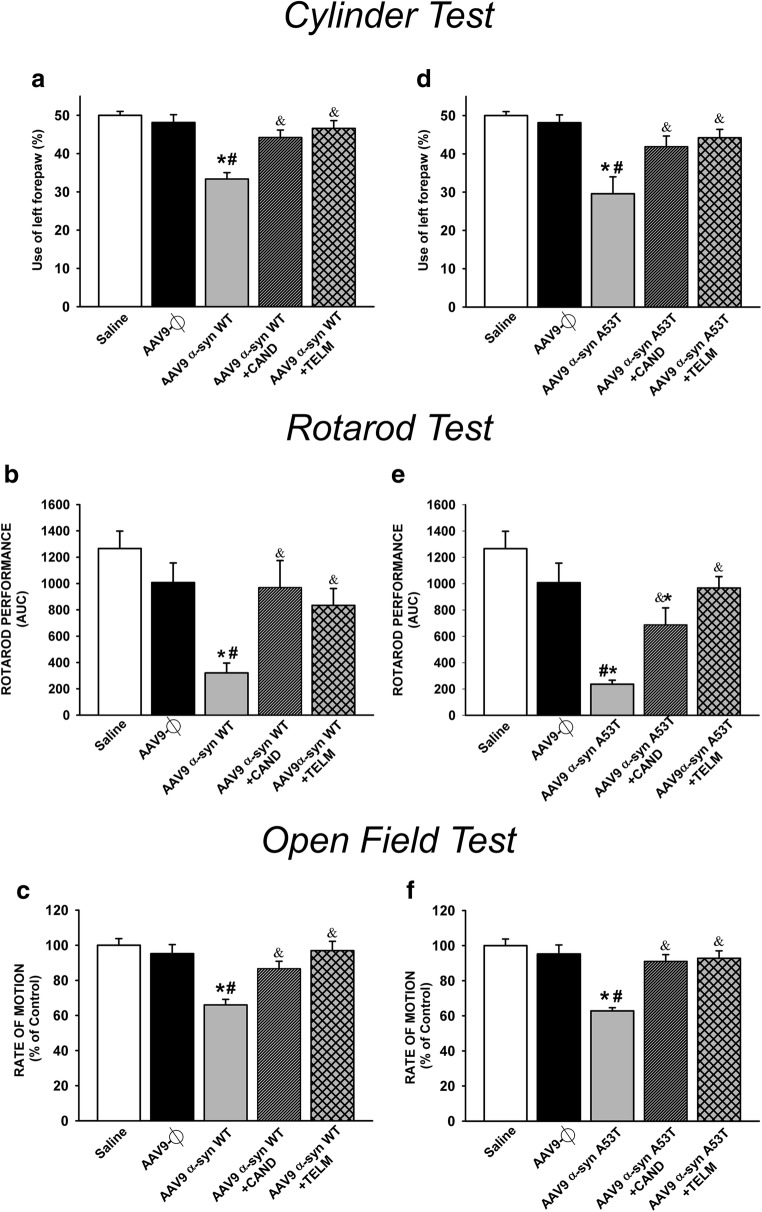
Behavioral analysis of rats in different experimental groups 4 weeks after injection of AAV9 vectors. Motor performance was analyzed using the cylinder test (a, d), the rotarod test (b, e), and the open field test for locomotor activity (c, f). Measurements show significant reductions in motor performance in lesioned rats relative to controls (saline and empty vectors), which were significantly improved by treatment with candesartan or telmisartan. Data are means ± SEM. ^*^p < 0.05 relative to saline-injected group; ^#^*p* < 0.05 relative to the AAV9-Ф-injected group; ^&^*p* < 0.05 relative to the group injected with AAV9 expressing human WT α-syn (a–c) or expressing human A53T mutated α-syn (d–f). One-way ANOVA followed by Holm–Sidak post hoc test. Abbreviations: α-syn A53T = A53T mutated alpha-synuclein; α-syn WT = wild-type alpha-synuclein; ANOVA = analysis of variance; CAND = candesartan; SEM = standard error of the mean; TELM = telmisartan; Ф = empty-null

In the cylinder test, rotarod test, and open field locomotor activity, rats injected with AAV9-WT α-syn (Fig. 7a–c) and rats injected with AAV9-A53T α-syn (Fig. 7d–f) showed a significant decrease in spontaneous use of the impaired forepaw (Fig. 7a, d), rotarod performance (Fig. 7b, e), and locomotor activity (Fig. 7c, f), which were significantly improved in lesioned rats treated with candesartan or telmisartan.

### Microglial Response After Overexpression of WT or Mutated A53T Αlpha-Synuclein: Effect of AT1 Receptor Blockers

**Fig. 8:**
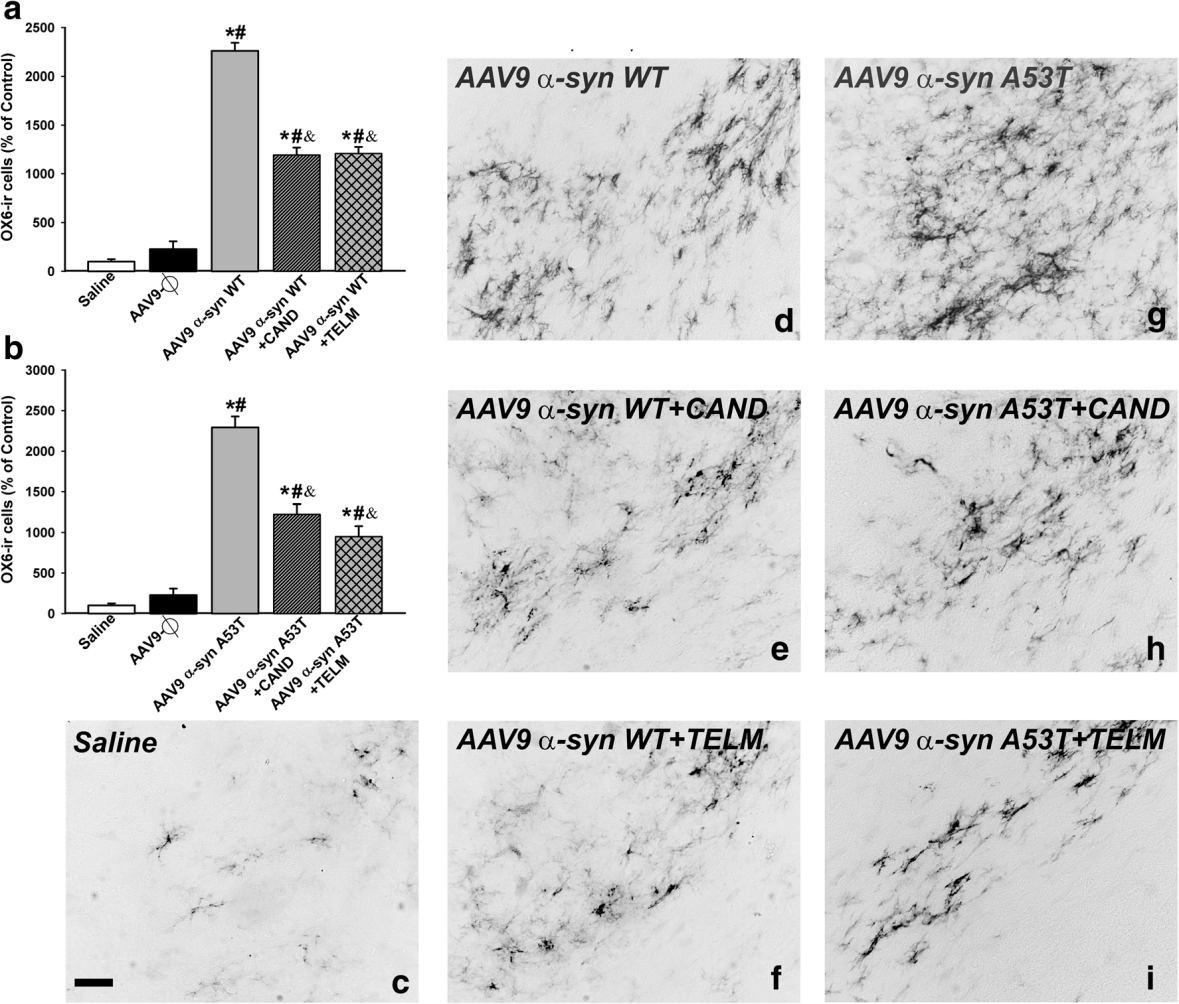
OX6 immunoreactive microglial cells in the substantia nigra compacta 4 weeks after injection of saline, or empty AAV9 vectors (AAV9-Ф), or AAV9 expressing human WT α-syn, or AAV9 expressing human A53T mutated α-syn in rats not treated or treated with the AT1 antagonist candesartan (CAND) or telmisartan (TELM). Representative photomicrographs of the substantia nigra compacta of different groups of rats are shown in (c)–(i). The estimated number of OX6-ir cells in the substantia nigra of the different experimental groups is shown in (a) and (b). The microglial cells are shown as % of that observed in controls (i.e., saline-injected rats), and data are means ± SEM. ^*^p < 0.05 relative to the group treated with saline, ^#^*p* < 0.05 relative to the AAV9-Ф-injected group, ^&^*p* < 0.05 relative to the group injected with AAV9 expressing human WT α-syn (a) or expressing human A53T mutated α-syn (b). One-way ANOVA and Holm–Sidak post hoc test. Scale bar = 100 μm. Abbreviations: ANOVA = analysis of variance; SEM = standard error of the mean; Ф = empty-null

**Fig. 9:**
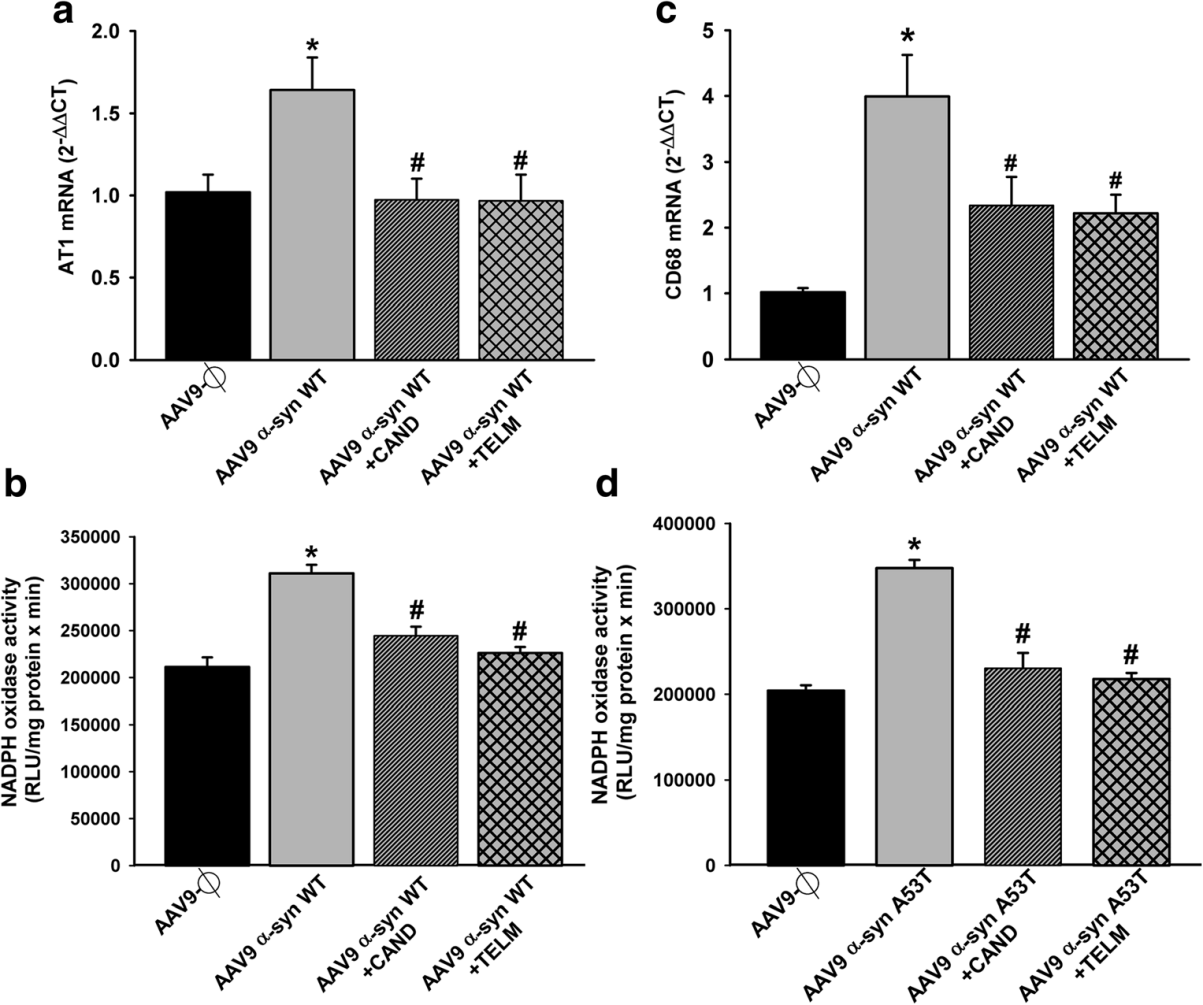
Effect of empty AAV9 vectors (AAV9-Ф) or AAV9 expressing human α-syn on the expression of AT1 receptor mRNA, NADPH activity, and markers of phagocytic activity (CD68) in rats not treated or treated with the AT1 antagonist candesartan (CAND) or telmisartan (TELM). AAV9 expressing human WT α-syn induced a significant increase in the expression of AT1 receptor mRNA (a), NADPH activity (b, d), and CD68 (c). A significant increase in NADPH activity induced by AAV9 expressing human A53T α-syn is shown in (d). These changes were inhibited by simultaneous treatment with the AT1 blockers candesartan or telmisartan (a–d). In (a) and (c), the results were normalized to the values of the control group. For RT-PCR, the comparative cycle threshold values method (2^−ΔΔCt^) was used. Gene expression was measured relative to that of the housekeeping transcripts (β-actin). NADPH oxidase activity was expressed as relative light units (RLU)/min/mg protein). Data are means ± SEM. ^*^p < 0.05 relative to the AAV9-Ф-injected group; ^#^*p* < 0.05 relative to the group injected with AAV9 expressing human α-syn. One-way ANOVA followed by Holm–Sidak post hoc test. Abbreviations: α-syn A53T = A53T mutated alpha-synuclein; α-syn WT = wild-type alpha-synuclein; ANOVA = analysis of variance; AT1 = angiotensin type 1 receptor; CAND = candesartan; CD68 = cluster of differentiation 68; SEM = standard error of the mean; TELM = telmisartan; Ф = empty-null

**Fig. 10:**
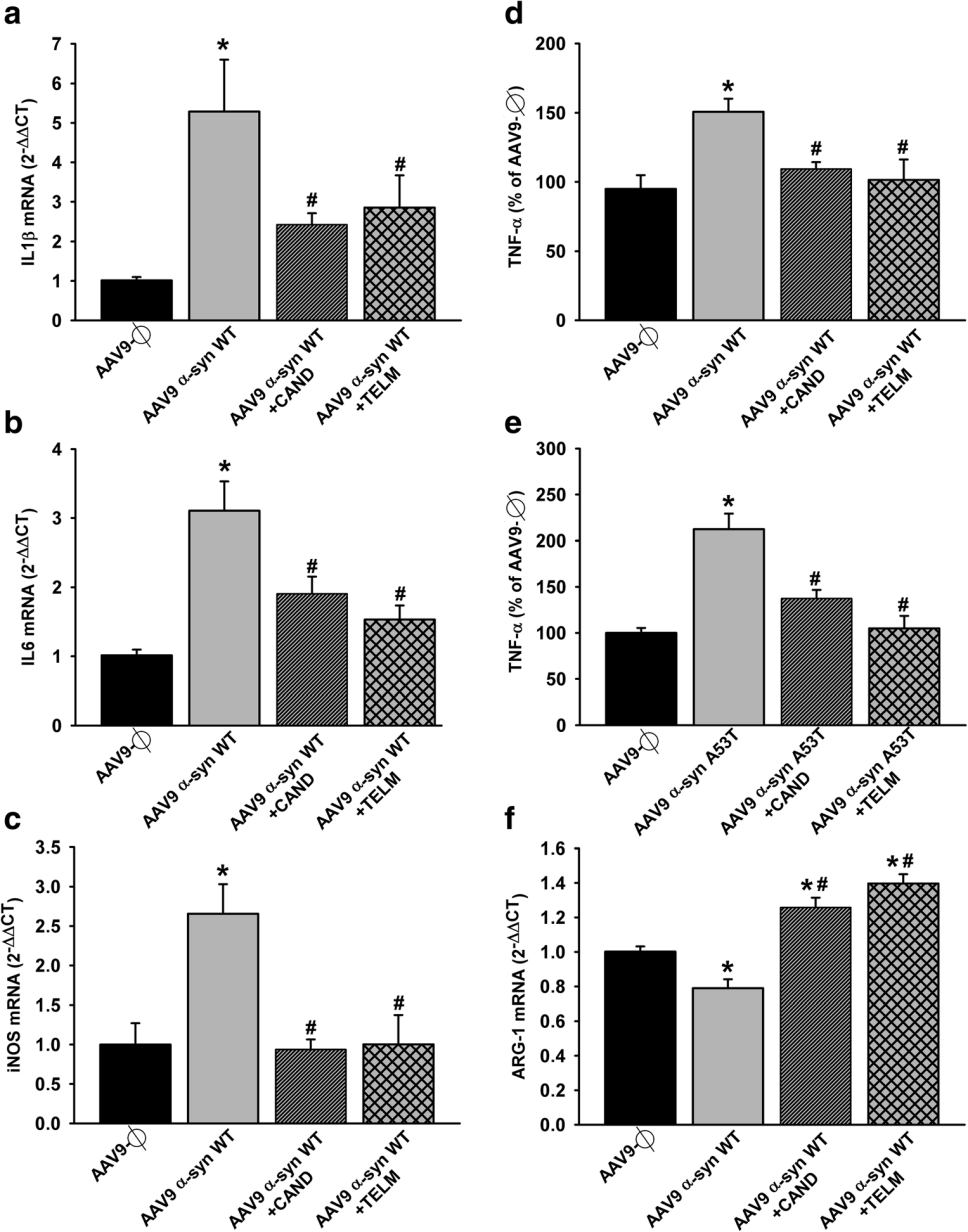
Effect of empty AAV9 vectors (AAV9-Ф) or AAV9 expressing human α-syn on the expression of markers of the microglial phenotype in rats not treated or treated with the AT1 antagonist candesartan (CAND) or telmisartan (TELM). AAV9 expressing human WT α-syn induced a significant increase in the expression of markers of the M1 cytotoxic phenotype (IL-1β, IL-6, iNOS, and TNF-α; a–d). The marked increase in TNF-α expression induced by AAV9 expressing human A53T α-syn is shown in (e). However, a decrease in the expression of the M2 repair/regenerative phenotype marker ARG-1 was observed (f). These changes were inhibited by simultaneous treatment with the AT1 blockers candesartan or telmisartan (a–f). The results were normalized to the values of the control group. For RT-PCR, the comparative cycle threshold values method (2^−ΔΔCt^) was used. Gene expression was measured relative to that of the housekeeping transcripts (β-actin). The levels of TNF-α were quantified with rat specific enzyme-linked immunosorbent assay (ELISA) kits in picograms per milliliter protein and expressed as percentages of the content in the control group samples. Data are means ± SEM. ^*^p < 0.05 relative to the AAV9-Ф-injected group; ^#^*p* < 0.05 relative to the group injected with AAV9 expressing human α-syn. One-way ANOVA followed by Holm–Sidak post hoc test. Abbreviations: α-syn A53T = A53T mutated alpha-synuclein; α-syn WT, wild-type alpha-synuclein; ANOVA = analysis of variance; ARG-1 = arginase-1; CAND = candesartan; IL-1β = interleukin-1β; IL-6 = interleukin 6; iNOS = inducible nitric oxide synthase; TELM = telmisartan; TNF-α = tumor necrosis factor alpha; SEM = standard error of the mean; Ф = empty-null

One week after injection of AAV9-WT α-syn or AAV9-A53T α-syn, the loss of dopaminergic neurons was not significant. However, an increase in density of Iba-1-ir cells was observed in the nigral region (Fig. 1h, k). Clear phagocytic phenotypes were uncommon, particularly phagocytic microglia engulfing neurons or syn-positive material. Four weeks after injection of AAV9-WT α-syn or AAV9-A53T α-syn, the loss of dopaminergic neurons was accompanied by a marked increase in microglial response as observed with Iba-1 labeling and confocal microscopy (Fig. 2b, e). Phagocytic microglial phenotypes were abundant and frequently showed engulfed syn-positive material (Fig. 2g–k). The high number of morphological phenotypes of classically activated microglia was confirmed with immunohistochemistry for OX6 (MHC class II). Rats injected with AAV9-WT α-syn or AAV9-A53T α-syn showed significantly higher number of OX6-ir cells than controls or rats injected with empty vectors. However, the effects of α-syn on the microglial response were significantly lower in rats treated with the AT1 blockers candesartan or telmisartan (Figs. 8, 9, and 10).

After observing the inhibitory effects of AT1 blockers on dopaminergic cell death and microglial response induced by α-syn overexpression, we investigated the effects of neuronal α-syn overexpression on the RAS pro-oxidative pro-inflammatory axis (Ang II/AT1/NADPH oxidase). Rats that overexpressed α-syn showed an increase in the expression of AT1 receptors and NADPH activity in the nigral region in comparison with control rats injected with empty-null vectors, and this increase was inhibited in rats treated with candesartan or telmisartan (Fig. 9a, b, d). Consistent with the abovementioned observations, rats injected with AAV9-α-syn showed a significant increase in the expression of the marker of phagocytic activity CD68 (Fig. 9c) and M1 microglial phenotype makers (iNOS and TNF-α, IL-1β, and IL-6) (Fig. 10a–e) and a significant decrease in the expression of the M2 microglial phenotype marker ARG-1 (Fig. 10f). These changes in microglial phenotype markers were inhibited by simultaneous treatment with the AT1 blockers candesartan or telmisartan (Fig. 10a–f).

## Discussion

In the present study, we observed that the AAV9-synapsin-α-syn vector led to overexpression of α-syn in dopaminergic neurons of the SNpc, which induced a marked dopaminergic neuron loss together with a concomitant microglial neuroinflammatory response. A similar level of dopaminergic cell death was observed 4 to 5 weeks after the vector injection in other recent models using efficient AAV vector constructs [13, 14]. A 10% additional increase in cell death was observed over the following 4 weeks in some studies [13]. However, early time points (i.e., 1 and 4 weeks after injection) are more adequate to study the direct α-syn effects on the microglial response than more chronic or advanced stages. We injected rats with AAV9 expressing WT or A53T mutated α-syn and both led to marked neuron death and microglial activation. In preliminary pilot experiments and consistent with previous studies [39], we observed more toxic effects with the A53T mutated α-syn constructs compared with WT α-syn. As a consequence, a lower vector dose was used for A53T mutated α-syn to get results more similar to that observed with WT α-syn.

Neuroinflammation is a major factor for progression of dopaminergic degeneration, and it has been suggested that the microglial neuroinflammatory response may be a major mechanism of α-syn toxicity [2, 6]. Consistent with this, an intense neuroinflammatory response has been observed in the present and previous studies using AAV viral vectors to induce α-syn overexpression [15, 16]. On this basis, the present study has been focused on the α-syn-mediated microglial response and, particularly, its possible modulation by AT1 receptor antagonists.

Several *in vitro* studies have observed that extracellular α-syn can induce microglial activation and cause neuron damage [4, 40, 41]. Interestingly, we observed a significant microglial response 1 week after AAV vector injection (i.e., before the observation of a significant neuron death), which suggests that intraneuronal α-syn accumulation may trigger the microglial response before neuron death. It is likely that signals derived from neurons altered by α-syn accumulation or α-syn released by neurons independently of cell death [42] initiate the microglial response. At later stages, a direct effect of the extracellular synuclein released from degenerated neurons can further activate microglia. In addition, microglia-mediated neuroinflammation and α-syn–induced neuronal damage may stimulate each other in a positive feedback self-perpetuating progression of neurodegeneration. The mechanisms underlying microglial activation have not yet been totally clarified. However, activation of the microglial response by lesioned neurons has been shown in numerous studies [43, 44]. Furthermore, several *in vitro* studies have observed that α-syn may activate Toll-like receptors of microglial cells and stimulate different microglial activation pathways, including the NF-κB and mitogen-activated protein kinase pathways (for review, see Zhang et al. [6]). It was also shown that α-syn induces microglial migration [45]. Interestingly, it has been observed that microglial NADPH oxidase (PHOX) is activated by the α-syn [38, 39]. Activation of NADPH oxidase induces superoxide release and plays a pivotal role in microglial polarization to a pro-inflammatory phenotype, which can be inhibited by NADPH oxidase depletion or inhibition [46, 47]. The observation of NADPH activation by α-syn is of particular interest for the present study, because it is well known that the pro-inflammatory axis of the RAS (Ang II/AT1) is a major activator of the NADPH oxidase complex in different cell types, and particularly in inflammatory cells such as microglial cells [18, 48]. Pro-inflammatory signals increase microglial AT1 receptor expression and activity [49], and Toll-like receptors, which are known to mediate classical microglial activation, may interact with AT1 receptors [50, 51]. Furthermore, we have recently shown an important interaction between RAS, NADPH oxidase, and Rho-kinase (ROCK) in microglia [18, 52, 53]. It is known that RhoA/ROCK is an important regulator of the actin cytoskeleton, which is particularly important for migration of inflammatory cells, including microglia [54], into inflamed areas and for several changes involved in phagocytosis [55]. However, the relationship between α-syn and microglial phagocytosis has been controversial, and both positive and negative effects have been observed *in vitro* [56, 57], which may be a consequence of different forms of α-syn and experimental conditions used [58]. In the present study, we observed evident phagocytosis of α-syn by microglial cells 4 weeks after AAV9-α-syn injections.

In the present *in vivo* model of overexpression α-syn, using an effective AAV9-α-syn vector that restricts transgene expression exclusively to neurons, we have shown that both neuronal WT and A53T mutated α-syn lead to dopaminergic neuron death and intense microglial pro-inflammatory response. We observed a marked increase in the number of OX6-positive microglial cells together with an increase in the expression of the marker of phagocytic activity CD68 and classical pro-inflammatory/M1 microglial phenotype markers such as iNOS, TNF-α, IL-1β, and IL-6 [59], and a significant decrease in the expression of markers of immunoregulatory/M2 microglial phenotype such as the enzyme ARG-1 [59, 60]. Interestingly, an inhibition of the microglial response led to a significant decrease in dopaminergic neuron death, which confirms in the present *in vivo* model a significant role of the microglial response in α-syn-induced dopaminergic neuron death and that modulation of the microglial response may be a useful therapeutical strategy. In the present study, we also showed that the brain RAS plays a major role in the α-syn-induced microglial response and α-syn-induced dopaminergic neuron death.

In several previous studies, we have shown the presence of a local paracrine RAS in neurons and glial cells in the substantia nigra and striatum of rodents and primates, including humans [61, 62]. This local RAS appears normally involved in the regulation of dopamine levels [63–65], modulation of intraneuronal levels of oxidative stress [66–68], and modulation of the neuroinflammatory response [18, 69]. In microglial cells, Ang II, via AT1 receptors, is a major activator of the microglial NADPH oxidase complex and microglial polarization toward the M1 pro-oxidative pro-inflammatory phenotype [17, 18] and modulates levels of microglial pro-inflammatory cytokines (such as TNF-α) and ROCK activity [52, 53, 70]. In the present *in vivo* study, we observed that overexpression of neuronal α-syn leads to increased expression of AT1 and NADPH oxidase activity together with an increase in microglial activation toward the M1 pro-inflammatory phenotype. Consistent with this, administration of AT1 blockers such as candesartan and telmisartan led to a significant decrease in the number of OX6-ir microglial cells, expression of CD68 mRNA, NADPH activity, expression of M1 phenotype markers, and α-syn-induced dopaminergic neuron death. Candesartan and telmisartan are the most effective AT1 blockers at crossing the blood–brain barrier, and low doses not affecting blood pressure are able to induce effects on brain RAS *in vivo* [71, 72] and *in vitro* [52, 73]. In addition to the inhibition of the Ang II/AT1/NADPH oxidase axis, candesartan and telmisartan may protect against dopaminergic neuron death by additional mechanisms. AT1 antagonists, particularly telmisartan, may lead to activation of the anti-inflammatory PPAR-γ receptors by a double mechanism that involves a pharmacological AT1-independent PPAR-γ agonistic effect and a direct effect of the blockage of the AT1 itself, which also induces PPAR-γ activation [24, 74]. Regulation of α-syn levels and neurotrophic factors (BDNF and GDNF) have also been suggested as additional mechanisms involved in telmisartan-mediated neuroprotection [75, 76]. The present results reveal that inhibition of the microglial activation is a major mechanism involved in the neuroprotective effect of candesartan and telmisartan on the dopaminergic cell death induced by α-syn overexpression. Our results suggest the repurposing of candesartan and telmisartan as neuroprotective strategy for PD.

## Electronic Supplementary Material

ESM 1 (PDF 112 kb)
